# Editorial: Immunonutrition: bridging precision nutrition and modern medicine

**DOI:** 10.3389/fnut.2025.1685397

**Published:** 2025-08-29

**Authors:** Jani Almeida, Helena Sá, Francisco José Pérez-Cano, Sofia Viana

**Affiliations:** ^1^Laboratory of Immunology and Oncology, Center for Neurosciences and Cell Biology (CNC), University of Coimbra, Coimbra, Portugal; ^2^Nephrology Department, Centro Hospitalar e Universitário de Coimbra, ULS Coimbra, Coimbra, Portugal; ^3^Physiology Section, Department of Biochemistry and Physiology, Faculty of Pharmacy and Food Science, University of Barcelona, Barcelona, Spain; ^4^Nutrition and Food Safety Research Institute (INSA-UB), Santa Coloma de Gramenet, Spain; ^5^Faculty of Medicine, Institute of Pharmacology and Experimental Therapeutics, University of Coimbra, Coimbra, Portugal

**Keywords:** immunonutrition, natural bioactive compounds, nutritional indices, clinical interventions, pre-clinical models

Inflammation imposes substantial metabolic demands and depletes critical nutrient reserves, often impairing immune function. While adequate nutrition is foundational to immune homeostasis, the targeted use of supraphysiological doses of immunomodulatory nutrients to redirect immune responses toward tolerance or resolution of inflammation remains an evolving frontier. Immunonutrition, a key domain within precision nutrition, encompasses bioactive compounds—nutrients and non-nutrients—such as amino acids, fatty acids, nucleotides, vitamins, minerals, polyphenols, glucans, and an expanding repertoire of pre-, pro-, sym-, and post-biotics. Its clinical applications span early-life immune development, cancer and infection management, modulation of autoimmunity and allergies, and attenuation of immunosenescence and inflamm-aging in chronic diseases. Despite the advances propelled by multi-omics research, critical gaps remain in mechanistic understanding, immune–nutrient interactions, synergy of bioactives within the food, biomarker identification, and the safety profile of immunonutrients, including nutrient–drug interactions. The nine contributions to this Research Topic advance immunonutrition across domains ranging from epidemiology and theoretical modeling to clinical intervention, illustrating the field's maturation and translational promise.

Four epidemiological studies based on NHANES datasets explore the predictive utility of composite nutritional–inflammatory indices. Ma et al. demonstrated that a higher Magnesium Depletion Score (MDS) independently predicted increased incidence and mortality from osteoarthritis, including cardiovascular mortality. These findings underscore magnesium as a modifiable immunonutrient in inflammation-driven degenerative disease. In patients with COPD, Yao et al. found a non-linear association between the Advanced Lung Cancer Inflammation Index (ALI) and mortality, with protective effects up to a threshold (~88–90), beyond which benefits declined. This U-shaped relationship highlights the nuanced role of nutritional and inflammatory balance in chronic respiratory disease outcomes. Xiao et al. introduced the Dietary Index for Gut Microbiota (DI-GM) and found it inversely associated with chronic kidney disease. Higher scores, driven by intake of fiber, whole grains, and coffee, were particularly protective in women, emphasizing the importance of sex-specific immunonutrition and the gut–immune–renal axis. Xu et al. evaluated the Dietary Inflammatory Index (DII) in over 43,000 adults and found that a higher score was independently associated with increased risk of coronary heart disease. These effects were mediated by metabolic and lipid-related pathways including BMI, triglyceride-glucose index, and HDL levels. Notably, the association was stronger in younger individuals, women, and those with otherwise low cardiovascular risk, suggesting that dietary inflammation may disproportionately affect populations traditionally considered at low-risk. Key dietary components included carbohydrates, vitamin C, and iron. Collectively, these studies validate composite dietary indices as both predictors and potential indicators of immune-mediated disease risk, while reinforcing the gut–immune–metabolic interface as a pivotal therapeutic axis.

Mechanistic innovation in immunonutrition is reflected in contributions that provide both conceptual and experimental tools. Ramal-Sanchez et al. developed a tri-cellular *in vitro* intestinal model incorporating enterocytes, goblet cells, and immune cells to simulate the human gut epithelium. Using this model, they evaluated the immunomodulatory effects of broccoli digesta and observed suppression of inflammatory cytokines (IL-6, TNF-α, IL-8, IL-18) alongside upregulation of tight junction proteins (ZO-1), supporting the model's utility for functional screening of food-derived immunonutrients. LeGrand et al. proposed the conceptual framework of “immune stressing,” in which effector immune cells impose metabolic stress, via glucose deprivation, ROS generation, and lactate accumulation, on pathogens. This resource-restriction model reframes nutritional immunity as an active metabolic strategy that targets the fragility of proliferating pathogens while sparing host cells. It extends the classic paradigm of micronutrient withholding and offers new avenues for understanding host–pathogen dynamics.

Three intervention studies further illustrate the translational potential and limitations of immunonutrition. Singh et al. conducted a longitudinal pediatric study to assess the neutropenic diet in children undergoing chemotherapy. Despite moderate adherence, the diet did not reduce febrile neutropenia, infections, or mortality. Socioeconomic status influenced compliance, and the findings suggest that restrictive dietary practices may not be necessary in this population. Instead, emphasis should shift toward food safety and hygiene. Qin et al., using a murine model of peritonitis, demonstrated that retinoic acid (RA) enhanced peritoneal macrophage phagocytosis, promoted recruitment of small peritoneal macrophages, and upregulated adhesion and migration gene expression. Encapsulation of RA in ZIF-8 nanoparticles sustained these effects, highlighting a promising strategy for precision-targeted immunonutrition, though further validation is needed for model specificity and delivery system stability. In a 90-day clinical pilot trial, Perlmutter et al. evaluated a polyphenol-rich Tartary buckwheat supplement in healthy adults. The intervention directionally influenced CpG methylation patterns across biological aging clocks (PCPhenoAge, PCGrimAge, OmicAge) and correlated with shifts in immune cell composition, particularly markers of immunosenescence. Pathway analyses highlighted ceramide kinase and immune regulatory networks, supporting polyphenols as modulators of epigenetic aging via immune-related mechanisms.

Several converging insights emerge across these contributions. Composite indices such as MDS, ALI, DI-GM, and DII show dual utility as biomarkers for disease risk and as intervention targets. Mechanistic innovation, through both modeling and conceptual frameworks, expands our understanding of how nutrients shape immune responses. Clinical interventions underscore both the promise of targeted strategies—such as polyphenols and RA nanoparticles—and the limitations of traditional approaches like exclusionary diets. Sex and life stage repeatedly emerge as key modifiers of nutritional immunity, highlighting the need for personalization. Multi-omics and systems-level analyses offer powerful tools for biomarker discovery and may help accelerate clinical translation. Nonetheless, persistent challenges include the cross-sectional design of most epidemiological studies, limited sample sizes in clinical trials, and the need for further refinement and validation of mechanistic models in human systems.

This Research Topic presents a cohesive body of work that advances immunonutrition from theory and mechanism to intervention and epidemiology. Through the development of nutritional indices, modeling of host–pathogen metabolic interactions, and testing of food-derived bioactives, these studies underscore the transformative potential of immunonutrition in modern medicine. Future efforts should prioritize longitudinal, multi-omic, and personalized approaches to fully integrate immunonutrition into evidence-based clinical practice. The editors thank all authors and reviewers for their contributions and invite continued interdisciplinary collaboration to propel the field forward.

